# Supplementary material for: Taste Sensitivity of Elderly People Is Associated with Quality of Life and Inadequate Dietary Intake
Source: Nutrients. 2021 May 17;13(5):1693. doi: 10.3390/nu13051693 (PMC8155931; doi:10.3390/nu13051693)
Supplement: Supplementary file 1 [file nutrients-13-01693-s001.zip › nutrients-1195506-supplementary.pdf]

Supplementary Table S1. Comparison of nutrient intake versus Dietary Reference Intake (DRI) between men and women and percentage of elderly with less than 75% nutrient intake compared to DRI in elderly group

	Nutrient intake in comparison to Dietary Reference Intake (%)				Percentage of elderly with nutrient intake under 75% DRI			
	Total (n=68)	Men (n=34)	Women (n=34)	*p*-value (Men vs Women)	Total (n=68)	Men (n=34)	Women (n=34)	*p*-value (Men vs Women)
Energy	72.3 ±30.5[a]	77.5 ±31.2	67.1 ±29.5	0.166	36 (52.9)[b]	15 (44.1)	21 (61.8)	0.224
Protein	98.2 ±52.4	105.4 ±54.5	91.1 ±50	0.265	28 (41.2)	11 (32.4)	17 (50.0)	0.218
Fiber	90.5 ±47.3	93.8 ±47.2	87.1 ±47.9	0.562	31 (45.6)	15 (44.1)	16 (47.1)	1.000
Vitamin A	56.4 ±42.4	66.1 ±46.4	46.7 ±36.1	0.058	51 (75.0)	23 (67.6)	28 (82.4)	0.262
Vitamin C	90.7 ±72.5	102.5 ±75.2	79 ±68.7	0.184	34 (50.0)	16 (47.1)	18 (52.9)	0.809
Thiamin	146.7 ±73.4	150.8 ±68.1	142.7 ±79.2	0.651	13 (19.1)	4 (11.8)	9 (26.5)	0.217
Riboflavin	94.1 ±53.6	100.7 ±55.2	87.5 ±51.9	0.315	32 (47.1)	16 (47.1)	16 (47.1)	1.000
Niacin	83.5 ±46.7	92.8 ±49.2	74.2 ±42.7	0.101	32 (47.1)	14 (41.2)	18 (52.9)	0.466
Vitamin B_6	120.9 ±110.6	141.2 ±129.5	100.6 ±84.8	0.131	26 (38.2)	10 (29.4)	16 (47.1)	0.212
Folic acid	115 ±64.4	131.4 ±65.1	98.6 ± 60.2	0.035	22 (32.4)	6 (17.6)	16 (47.1)	0.019
Vitamin B_{12}	376.2 ±316.4	441.6 ±363.6	310.7±249.5	0.088	4 (5.9)	1 (2.9)	3 (8.8)	0.614
Calcium	60.9 ±42.8	75.3 ±47.8	46.6 ±31.8	0.005	48 (70.6)	21 (61.8)	27 (79.4)	0.183
Phosphorus	130.9 ±73	150.3 ±79.6	111.6 ±61.1	0.028	20 (29.4)	6 (17.6)	14 (41.2)	0.061
Sodium	259 ±166.3	313.6 ±183.4	204.3 ±127.9	0.006	3 (4.4)	0 (0.0)	3 (8.8)	0.239
Iron	180 ±91.5	188.4 ±86.5	171.5 ±96.8	0.450	9 (13.2)	4 (11.8)	5 (14.7)	1.000
Zinc	130.4 ±75.7	133.3 ±78.8	1275. ±73.5	0.756	17 (25.0)	7 (20.6)	10 (29.4)	0.576

[a] mean ± standard deviation (SD), [b] n (%)

Supplementary Table S2. Reciprocal causalities of taste thresholds and QOL of elderly participants investigated by simultaneous equation models with three-stage least squares estimation

	Estimate	SE	t	p
Sucrose RT = β_1 QOL	-8.435	1.837	-4.592	<0.001
QOL = β_2 Sucrose RT	-0.116	0.011	-10.909	<0.001
NaCl RT = β_1 QOL	-8.582	1.446	-5.935	<0.001
QOL = β_2 NaCl RT	-0.104	0.018	-5.682	<0.001
Caffeine RT = β_1 QOL	-4.699	1.243	-3.779	<0.001
QOL = β_2 Caffeine RT	-0.067	0.030	-2.235	0.029
Citric acid RT = β_1 QOL	-8.951	1.858	-4.818	<0.001
QOL = β_2 Citric acid RT	-0.110	0.016	-7.034	<0.001
Umami RT = β_1 QOL	-7.579	2.689	-2.819	0.006
QOL = β_2 Umami RT	-0.144	0.023	-6.285	<0.001

In the equation, non-nutritional factors that were significantly related to QOL or taste RTs were used as instrumental variables. Significant reciprocal causalities between each taste RT and QOL were shown.
